# A self-report comorbidity questionnaire for haemodialysis patients

**DOI:** 10.1186/1471-2369-15-134

**Published:** 2014-08-18

**Authors:** Sivakumar Sridharan, Jocelyn Berdeprado, Enric Vilar, Justin Roberts, Ken Farrington

**Affiliations:** 1 Renal Unit Lister Hospital, Stevenage SG1 4AB, UK; 2 Health and Human Sciences Research Institute, University of Hertfordshire, Hatfield AL10 9AB, UK

**Keywords:** Comorbidity, Questionnaire, Haemodialysis, Survival, End-stage renal disease

## Abstract

**Background:**

Patients with end-stage renal disease (ESRD) have multiple comorbid conditions. Obtaining comorbidity data from medical records is cumbersome. A self-report comorbidity questionnaire is a useful alternative. Our aim in this study was to examine the predictive value of a self-report comorbidity questionnaire in terms of survival in ESRD patients.

**Methods:**

We studied a prospective cross-sectional cohort of 282 haemodialysis (HD) patients in a single centre. Participants were administered the self-report questionnaire during an HD session. Information on their comorbidities was subsequently obtained from an examination of the patient’s medical records. Levels of agreement between parameters derived from the questionnaire, and from the medical records, were examined. Participants were followed-up for 18 months to collect survival data. The influence on survival of comorbidity scores derived from the self-report data (the Composite Self-report Comorbidity Score [CSCS]) and from medical records data - the Charlson Comorbidity Index [CCI] were compared.

**Results:**

The level of agreement between the self-report items and those obtained from medical records was almost perfect with respect the presence of diabetes (Kappa score κ 0.97), substantial for heart disease and cancer (κ 0.62 and κ 0.72 respectively), moderate for liver disease (κ 0.51), only fair for lung disease, arthritis, cerebrovascular disease, and depression (κ 0.34, 0.35, 0.34 and 0.29 respectively). The CSCS was strongly predictive of survival in regression models (Nagelkerke R^2^ value 0.202), with a predictive power similar to that of the CCI (Nagelkerke R^2^ value 0.211). The influences of these two parameters were additive in the models – suggesting that these parameters make different contributions to the assessment of comorbidity.

**Conclusion:**

This self-report comorbidity questionnaire is a viable tool to collect comorbidity data and may have a role in the prediction of short-term survival in patients with end-stage renal disease on haemodialysis. Further work is required in this setting to refine the tool and define its role.

## Background

Patients with end-stage renal disease (ESRD) often have a number of comorbid conditions. Comorbidity is an important outcome measure in patients with ESRD and has been shown to be a significant predictor of mortality in this patient population [1-5]. Hence, obtaining information on comorbid conditions is vital for clinical and research purposes.

The Charlson Comorbidity Index (CCI) was developed to predict patient survival using comorbidity data in longitudinal studies [6]. The CCI has been shown to be a significant predictor of clinical outcomes in ESRD patients [4,7]. CCI is calculated from information gathered from medical records which is time-consuming. Moreover, data collection may be limited by the ease of availability of the records and the accuracy of the documentation of specific medical conditions.

A simple, self-report questionnaire would be a useful alternative tool for collecting comorbidity data. Self-report questionnaires derived from the general population may not be suitable for patient populations with specific chronic diseases such as ESRD. It is important to define the role of such questionnaires in disease-specific patient population. We developed a simple self-report questionnaire to obtain comorbidity data from patients with ESRD.

Our aim was to explore the level of agreement of the information obtained from the questionnaire and medical records. We also wished to study the association between the questionnaire-derived comorbidity score and short-term survival and relate this to the performance of the established Charlson Comorbidity Index (CCI).

## Methods

### Self-report questionnaire

The self-report comorbidity questionnaire (see Additional file 1) was based on that developed by Sangha et al. [8]. This questionnaire has been validated in general medical and surgical patients and had been shown to correlate with subsequent health status and resource utilisation [8]. We chose eight conditions that are commonly prevalent in patients with ESRD to include in the questionnaire. The conditions were expressed in a plain language that could be understood by individuals without any prior medical knowledge. Also, participants had the option of adding 3 additional medical conditions other than the ones that were listed. The first 2 questionnaire items, both enquired about cardiac disease, one relating to current and the other past history. This enabled more complete data capture regarding heart disease even in patients without ongoing symptoms.

As all study participants had ESRD and were receiving HD, we did not include the question about renal disease. For each of the listed conditions and the additional ones the participants could add, they were asked 3 questions – whether they had the condition, if they did, whether they were receiving any treatment and whether the disease was limiting their activities. Participants were asked to tick the respective box if the answer to the corresponding question was in the affirmative. The questions regarding treatment and limitation of activities served as a surrogate marker of the severity of the illness. All participants completed the questionnaire on their own without any help from healthcare staff.

The questionnaire was translated into Bengali and Urdu to facilitate data capture from different ethnic patients who did not have sufficient knowledge of English to complete the questionnaire. The translation was carried out by Straker Translations (London, UK). Two independent reviewers, who were native speakers of these languages, verified the accuracy of the translations. Copies of the translated versions of the questionnaire can be obtained by contacting the corresponding author.

### Scoring of the questionnaire

Each positive response had a score of 1. Hence, the maximum score was 3 for each medical condition – 1 for the presence of the disease, 1 for being on treatment and 1 if the disease was limiting their activities. Of the listed conditions, the first 2 referred to cardiac disease and were considered as one item for purposes of scoring. A judgement was made on the admissibility of the optional items as significant comorbidities. In the event of the additional items listed by patients (see later), we considered cerebrovascular disease (“stroke”) to be the only additional comorbidity with potential survival impact listed with sufficient frequency to merit inclusion in the scoring. In addition, since the level of agreement for depression between the questionnaire and the medical records was poorest of all listed conditions (see later) and since depression did not contribute to any of the models tested, this parameter was omitted from the scoring scheme. Hence the Composite Self-report Comorbidity Score (CSCS) was derived from 7 conditions – giving a potential maximum of 21. Age was not included in this score.

### Subjects and protocol

#### Ethical review

The study was approved by the North Wales Ethical Review Committee. All subjects gave informed written consent to take part.

#### Subjects

Patients on maintenance, in-centre HD were recruited from the Renal units of East and North Hertfordshire NHS Trust. The study included patients older than 18 years, dialysing 3 times a week and those able to understand English, Bengali or Urdu to allow them to complete the questionnaire. Exclusion criteria included patients with no capacity to consent, those dialysing other than 3-times weekly and those with limb amputations.

#### Study protocol

Each participant was administered the questionnaire on attendance for their regular HD session and asked to complete it by the end of that session. The demographic information of the participants (age, sex, and ethnicity) and their documented comorbidities were collected from our renal database. Our electronic renal database is continuously updated through inputs from clinician assessments and regular patient reviews. The Charlson Comorbidity Index (CCI) was calculated as previously described [6]. One of the authors (JB) administered and collected the questionnaire from the participants. Data from the medical records was extracted by one of the other authors (SS) without prior knowledge of individual comorbidity scores of the participants. All participants were followed-up for 18 months to obtain survival data.

### Statistical analysis

Statistical analysis was carried out using SPSS® version 19 (SPSS Software, IBM Corporation, Armonk, New York, USA). Normally distributed data are presented as mean ± SD, and non-normally distributed as median (interquartile range [IQR]). Correlations between scores were determined using the Spearman coefficient.

We assessed the agreement for individual items between the questionnaire and medical-record derived CCI using the inter-rater kappa (κ) statistic [9]. We also calculated the overall agreement, defined as the number of cases in which both the patient responses and medical records agreed (both “yes” and “no” responses) divided by the total number of cases.

We used Logistic regression models to identify independent predictors of survival in the study population. All models included age, sex, and ethnicity as variables. Individual patient-reported comorbid conditions that were significantly associated with survival were identified, as well as the contribution of the Composite Self-report Score (CSCS) and the CCI.

We constructed Receiver Operator Curves (ROC) to compare the utility of the CSCS and the CCI in predicting mortality as well as defining the optimal cut-off points for this prediction for both these scales. We compared the levels of agreement for the cut-off point in the scales using the inter-rater κ statistic [9]. In addition we compared the predictive power of these cut-off points in predicting mortality in Cox Regression models.

## Results

A total of 282 patients were recruited out of 350 haemodialysis patients in our unit over a period of 1 month. There were 177 males (62.8%). The mean age was 64.1 ± 15.3 years. The ethnic make-up was 201 white (71.3%), 51 South Asian (18.1%) and 30 black (10.6%) patients. For the open-ended questions, 46 patients (16.3%) indicated 1 additional disease, 10 patients (3.5%) indicated 2 diseases and 1 patient indicated 3 diseases. Frequently mentioned additional diseases included hypertension (11), stroke (10), hypothyroidism (5), visual impairment (5), and peripheral vascular disease (2). Of these only stroke was considered as significant additional comorbidity as discussed earlier. Hypertension was not included since this is a feature of chronic kidney disease and present in a very high proportion of patients on haemodialysis and Peripheral vascular disease since it was listed only twice. The other conditions mentioned by patients were hearing loss, back pain, inguinal hernia, insomnia, lymphoedema, gout, glaucoma and diverticulitis.

### Level of agreement

Table 1 shows the prevalence of each item as determined from the medical records and the comorbidity questionnaire and the level of agreement between them. Prevalence of heart disease, diabetes mellitus and cancer was similar between the medical records and the questionnaire. The prevalence of both lung and liver disease as obtained from medical records was higher than the self-reported. For the prevalence of arthritis and depression the opposite prevailed. Overall agreement exceeded 80% for all items with the highest agreement for diabetes mellitus (99%) and the lowest for arthritis (81%).

**Table 1:** Prevalence and levels of agreement of comorbid conditions according to Self-report comorbidity questionnaire and medical records

	**Prevalence (%)**			**Level of agreement (%)**	**Kappa (95% CI)**	**Interpretation of Kappa**
	**Records**	**Self-report**	**Limiting**			
**Diabetes**	84 (29.8)	82 (29.1)	22 (7.8)	99	0.97 (0.93, 1.00)	Almost Perfect
**Heart disease**	98 (34.8)	92 (32.6)	37 (13.1)	83	0.62 (0.52, 0.72)	Substantial
**Cancer**	20 (7.1)	18 (6.4)	3 (1.1)	96	0.72 (0.55, 0.89)	Substantial
**Liver disease**	12 (4.3)	7 (2.5)	3 (1.1)	97	0.51 (0.20, 0.83)	Moderate
**Arthritis**	22 (7.8)	72 (25.5)	47 (16.7)	81	0.35 (0.09, 0.50)	Fair
**Lung disease**	29 (10.3)	13 (4.6)	8 (2.8)	91	0.34 (0.10, 0.58)	Fair
**Cerebrovascular disease**	10 (3.5)	22 (7.8)	2 (0.7)	93	0.34 (0.09, 0.56)	Fair
**Depression**	17 (6.0)	35 (12.4)	15 (5.3)	88	0.29 (0.06, 0.51)	Fair

Variables considered were Age, Sex, Ethnicity, the presence of self-report Heart disease, Cerebrovascular disease, Cancer, Diabetes, Liver disease, Lung disease, and Arthritis.

Table 1 also shows the κ statistic and the interpretation of the κ for each item. There was almost perfect agreement between the two instruments for diabetes, substantial agreement for heart disease and cancer, and moderate agreement for liver disease. There was only fair agreement for lung disease, arthritis, depression and cerebrovascular disease. There was no association observed between gender and agreement of patient self-reports with medical records for any of the items listed. Using age as a dichotomous variable (age less than 65 vs. 65 or more) in this analysis, we found significant differences in level of agreement for arthritis and depression. κ was significantly higher in older patients for arthritis and in younger patients for depression (p = 0.03 for both).

### Composite comorbidity scores

**Figure 1:**
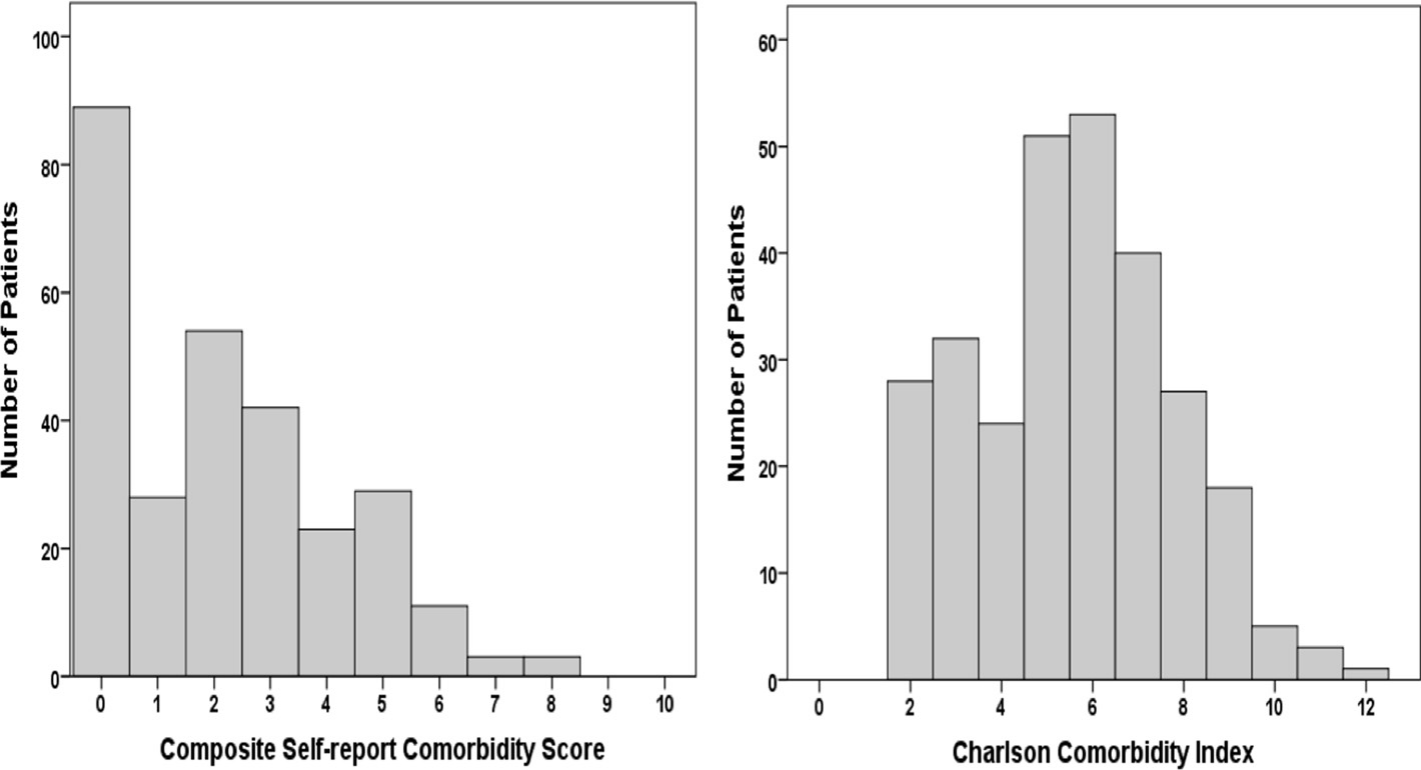
Histogram showing distribution of Composite Self-report Comorbidity Score (left hand panel) and Charlson Comorbidity Index (right hand panel).

The distributions of the CSCS and CCI are shown in Figure 1. The median CSCS was 2 (IQR3). The median CCI was 6 (IQR3). The CSCS correlated with the CCI (rho = 0.531; p < 0.001).

### Survival prediction

Of the 282 participants, 58 (20.6%) died in the 18 months following recruitment.

### Logistic regression

Table 2 shows the best Logistic Regression Model for predictors of survival at 18 months based on *individual* self-report comorbid conditions (Hosmer and Lemeshow Chi-square 11.115; p = 0.195: Nagelkerke R-square value 0.197). Variables considered were age, sex, ethnicity, presence of self-report heart disease, cerebrovascular disease, cancer, diabetes, liver disease, lung disease, and arthritis. Heart disease, liver disease and arthritis, along with age, were significant predictors of survival.

**Table 2:** The best Logistic Regression Model for predictors of survival at 18 months based on individual self-report comorbid conditions

	**B**	**S.E.**	**Wald**	**p-value**	**Exp(B)**
**Age**	.030	.013	5.287	.021	1.030
**Heart disease**	1.242	.319	15.124	.000	3.462
**Liver disease**	1.794	.856	4.392	.036	6.013
**Arthritis**	.858	.334	6.582	.010	2.358
**Constant**	−4.169	.902	21.374	.000	.015

The logistic regression models for the CSCS and the CCI are shown in Table 3. The models including these individual parameters showed similar goodness of fit (Hosmer and Lemeshow Chi-square 4. 151 [p = 0.843] and 1.878 [p = 0.985]) and predictive power (Nagelkerke R-square values 0.202 and 0.211 respectively). Each parameter had a highly statistically significant relationship to survival within the models (p <0.001 in both cases). Interestingly the model was improved by inclusion of both parameters (Nagelkerke R-square 0.250) and in this model (Table 3 – lower panel) both parameters retained a high degree of statistical significance. This suggests that each of these parameters contributes different aspects to the assessment of comorbidity.

**Table 3:** Logistic regression models for survival at 18 months

**MODEL 1: Nagelkerke R**^**2**^ **= 0.202**	**B**	**S.E.**	**Wald**	**p-value**	**Exp(B)**
Age (Years)	.030	.013	5.540	.019	1.031
Sex (Male v Female)	.440	.344	1.635	.201	1.553
Ethnicity (Non-white v White)	-.222	.381	.340	.560	.801
CSCS	.392	.081	23.386	.000	1.480
Constant	−4.616	.962	23.026	.000	.010
**MODEL 2: Nagelkerke R**^**2**^ **= 0.211**					
Age (Years)	-.015	.016	.894	.344	.985
Sex (Male v Female)	.211	.348	.367	.545	1.235
Ethnicity (Non-white v White)	-.213	.376	.322	.570	.808
CCI	.521	.105	24.445	.000	1.683
Constant	−3.641	.909	16.035	.000	.026
**MODEL 3: Nagelkerke R**^**2**^ **= 0.250**					
Age (Years)	-.002	.016	.021	.886	.998
Sex (Male v Female)	.298	.355	.702	.402	1.347
Ethnicity (Non-white v White)	-.315	.387	.663	.416	.730
CCI	.365	.117	9.715	.002	1.440
CSCS	.262	.092	8.180	.004	1.300
Constant	−4.251	.972	19.125	.000	.014

Model 1 includes the Composite Self-report Comorbidity Score (CSCS). Model 2 includes the Charlson Comorbidity Index (CCI). Model 3 includes both parameters.

### Receiver Operator Characteristics (ROC) analysis

**Figure 2:**
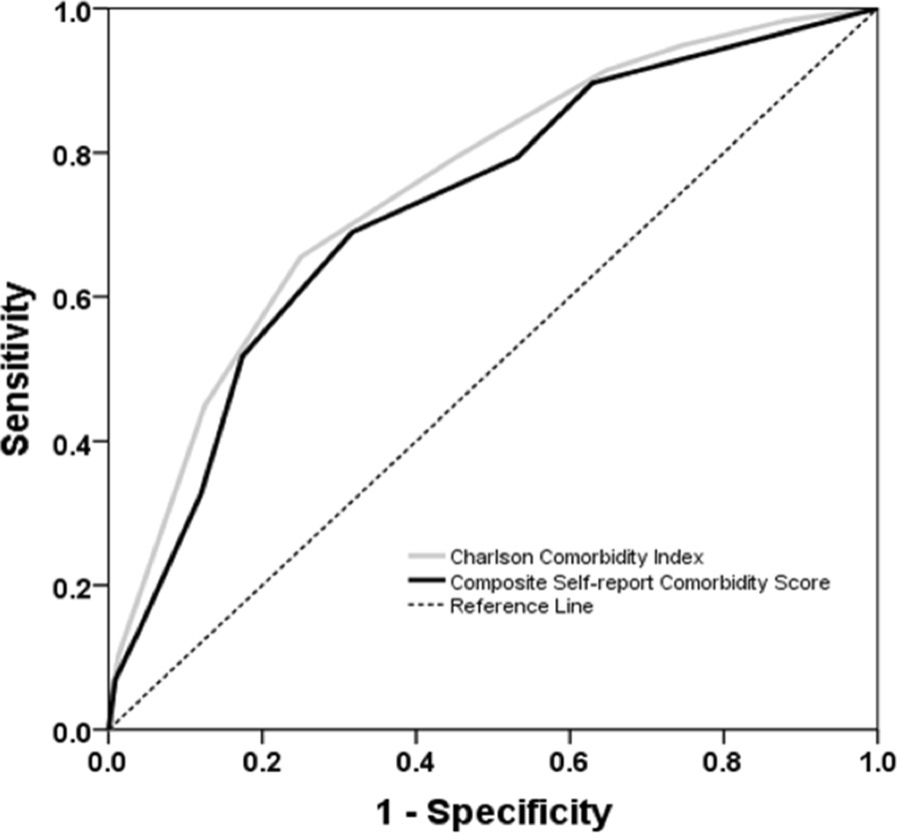
Receiver Operator Characteristic curves comparing compare the performance of the Composite Self-report Comorbidity Score and the Charlson Comorbidity Index in predicting death within the follow-up period.

We constructed ROC curves to compare the performance of CSCS and CCI in predicting death within the follow-up period, and to determine the best cut-off values of both these parameters for this prediction (Figure 2). The Area under the Curve in ROC analysis was similar for these parameters with overlapping 95% confidence intervals: 0.724 (0.651 – 0.797) and 0.754 (0.684 – 0.823) respectively. The best cut-off points for predicting mortality during the follow-up period were determined as CSCS >3 and CCI > 6. Patients with CSCS > 3 are subsequently referred to as having high CSCS. Likewise high CCI values refer to CCI > 6.

### Comparison of cut-off points of CSCS and CCI in mortality prediction

**Figure 3:**
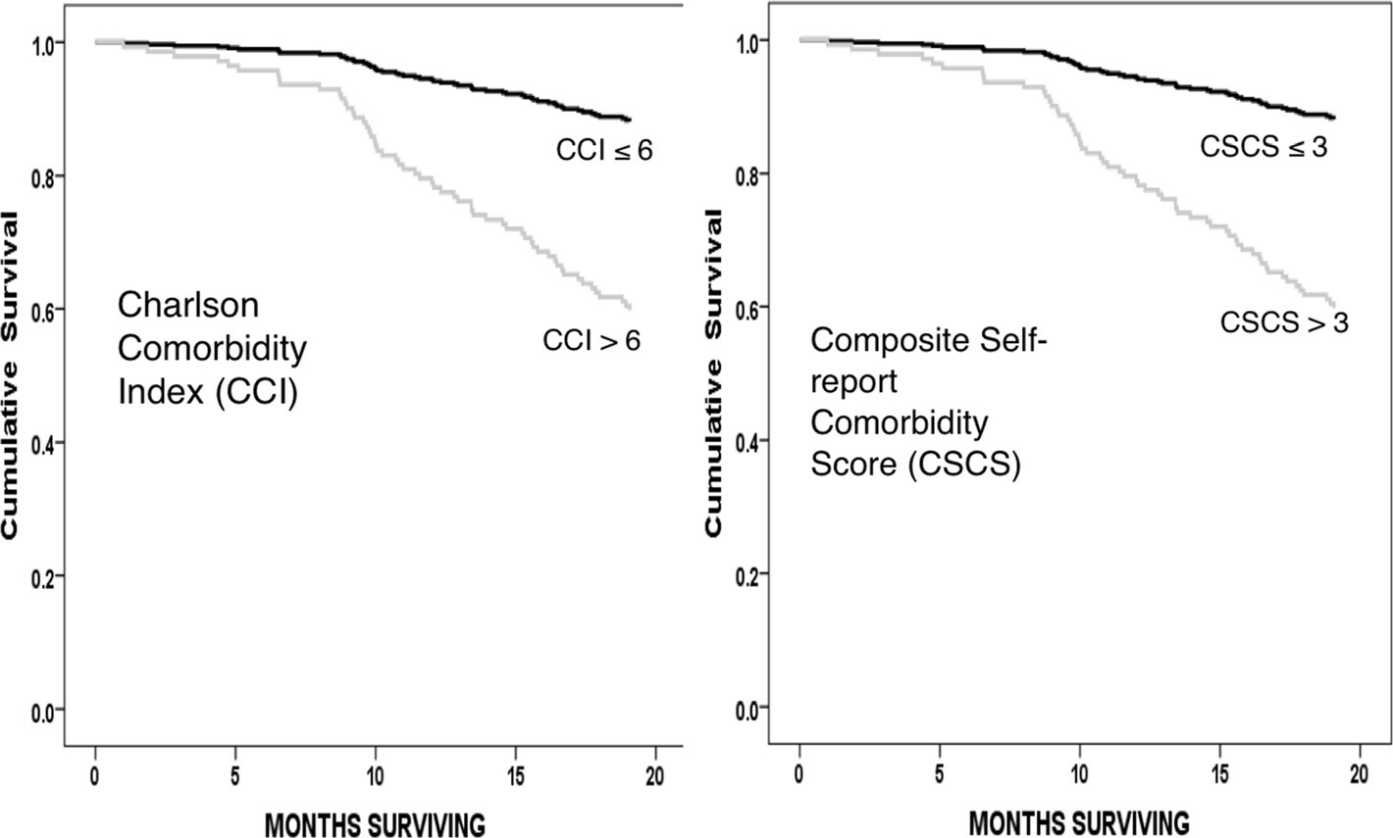
**Adjusted Survival for patients with high comorbidity scores (Charlson Comorbidity Index > 6 – Left panel and Composite Self-report Comorbidity Score > 3 – Right Panel).** Each Cox Regression model also included Age, Sex and Ethnicity.

Figure 3 compares the adjusted survival of patients with high CSCS (69 patients) and those with high CCI (94 patients). In both cases survival is adjusted for age, sex and ethnicity in Cox Regression models. Both high CSCS and high CCI were highly predictive of mortality within their separate models (p < 0.001 in both cases). Hazard Ratios were over 4 for both parameters with overlapping 95% Confidence Intervals (4.050 [3.362 – 6.947] and 4.139 [2.202 – 7.774] respectively). In spite of this, when these parameters were included together in the same Cox model, both retained their significance, and Hazard Ratios of each were similar, approaching 3 (Table 4). This again suggests that each parameter contributed different elements to the assessment of comorbidity. In keeping with this, the level of agreement between patient with high CSCS and high CCI was only fair (κ = 0.325; p < 0.001). The main difference between these high comorbidity groups relates to mean age which, unsurprisingly, was significantly higher in the high CCI group than in the high CSCS group (73.6 ± 8.7 vs. 67.4 ± 11.7; p <0.001). There were other differences. The best levels of agreement between CCI (high vs. low) and the presence or absence of individual components of the CCI, were for heart disease (κ 0.447), diabetes (κ 0.443), cerebrovascular disease (κ 0.301) and cancer (κ 0.205). On the other hand the best agreements between CSCS (high vs. low) and the presence or absence of individual components of the CSCS, were with heart disease (κ 0.491) and arthritis (κ 0.442) and diabetes (κ 0.396).

**Table 4:** Cox model of predictors of short term survival in haemodialysis patients

	**B**	**S.E.**	**Wald**	**p-value**	**Exp(B)**
**Age (Years)**	.011	.012	.840	.359	1.011
**Sex (Male v Female)**	.405	.299	1.831	.176	1.500
**Ethnicity (Non-white v White)**	-.411	.315	1.700	.192	.663
**CCI > 6**	1.020	.334	9.348	.002	2.773
**CSCS > 3**	1.067	.292	13.309	.000	2.907

The model contains both the Composite Self-report Comorbidity Score (CSCS) and the Charlson Comorbidity Index (CCI).

## Discussion

Information on comorbid conditions is essential in routine clinical practice and also for research purposes. We have designed a simple, self-report questionnaire to obtain comorbidity data in patients with advanced kidney failure, receiving treatment by dialysis. The questionnaire-derived comorbidity score – the CSCS – was significantly predictive of short-term survival in this patient group and may have clinical utility.

We found almost perfect or substantial levels of agreement between the prevalence of self-reported diabetes, heart disease and cancer and the prevalence of these conditions derived from the detailed examination of the patients’ medical records. The level of agreement for liver disease was moderate. For arthritis, lung disease, cerebrovascular disease and depression the levels of agreement were only fair. Whilst lung disease and cerebrovascular disease were under-reported by patients, arthritis and depression were reported more frequently compared to the medical records. There are a number of factors which may contribute to these discrepancies. Our data suggests that even though lung disease was under-reported in the questionnaire, a high proportion of those patients who did report having this condition indicated that their disease limited their activities. This suggests that patients with milder forms of lung disease may not attribute much significance to related symptoms (e.g. “smoker’s cough”) or may not be aware of the diagnosis at all. The under-reporting of cerebrovascular disease was almost certainly caused by the fact that this was not a condition specified on the questionnaire and patients wishing to report this condition had to write this in under “other medical conditions”. The conditions which were over-reported by patients, arthritis and depression, are predominantly diagnosed and treated in primary care. This may sometimes lead to their not being documented in hospital records unless they are receiving medication for that condition or if its severity warrants secondary care referral. The poorest level of agreement was found with depression and this, and its failure to contribute to any of the survival models we considered, caused us to exclude this parameter from contributing to the CSCS. Furthermore, though depression has been shown to be associated with mortality in CKD population, the diagnosis of depression in these studies has been made formally using physician diagnosis, clinical coding, or validated self-report screening tools, not by a single question in a self-report questionnaire the response to which may well just reflect subjects’ transient feelings.

We found that the CSCS was a significant predictor of survival in haemodialysis patients. The main individual comorbid conditions contributing to this predictive power in this patient group were the presence of heart disease, liver disease and arthritis (Table 2).

We compared the power of the CSCS and the CCI in predicting mortality over the 18 month follow-up period. In logistic regression models which allowed adjustment for age, sex and ethnicity, we found the parameters to have similar predictive power (Table 3). Since the CCI includes a term for age, such a modelling approach is required to allow for this in the comparison. The ROC analysis also showed that the parameters performed similarly in predicting death within the follow-up period. The area under the ROC curve was greater for CCI than for CSCS (0.754 vs. 0.724). This difference was not significant and is probably accounted for by the fact that age is not controlled for in this method of comparison.

We also used ROC analysis to help determine the best cut-off points for the CSCS and CCI for predicting mortality during follow-up. The high comorbidity groups were determined as CSCS >3 and CCI > 6. The performances of these parameters in predicting death were similar in Cox Models, again controlling for age, sex and ethnicity (Figure 3).

Interestingly the use of CSCS together with CCI in a logistic regression model improved the model with both terms retaining significant predictive power. The findings were similar with the use of both high CSCS and high CCI in a Cox Model. These findings, together with the only fair level of agreement between high CSCS and high CCI suggest that these parameters make different contributions to the assessment of comorbidity. The obvious difference relates to the inclusion of a contribution of patient age in the calculation of CCI. Indeed we found that high CCI group was significantly older than the high CSCS group. There were other differences between these two groups. Both were similarly influenced by their components relating to heart disease, and diabetes but the high CCI group was more influenced by components relating to cerebrovascular disease and cancer, and the high CSCS group by the component related to arthritis.

Instruments such as the CCI rely on availability and accuracy of medical records and as such, may be limited in its utility for clinical and research purposes. A self-report comorbidity questionnaire can help collect this information reliably and with relative ease. Various self-report health measures have been previously studied in patients with renal failure [10,11]. Our self-report questionnaire has the advantage of being brief, easily understandable by patients and at the same time being comprehensive enough to include commonly prevalent comorbid conditions in ESRD patient population. Also, the questionnaire enquires about the treatment and limitations imposed by specific diseases which can be used as a surrogate marker of the severity of the disease. The high levels of agreement between the self-report and clinical records with respect to diabetes, heart disease and cancer suggests that the utility of the self-report approach could embrace the collection of such condition-specific data for inclusion in survival models.

There are a number of limitations to our study. It has been shown previously that medical records may in themselves have substantial errors [12-14] and hence, using this method as a “gold standard” may be less than ideal. Also, with any questionnaire-based technique there is a potential for recall bias. Though patients had the option of adding any additional diseases that were not listed, it is possible that patients may not recall milder forms of existing comorbid diseases and this may exclude some important comorbid conditions such as cerebrovascular disease and peripheral vascular disease. Further development of this questionnaire should include specific enquiry about the presence and severity of these conditions. Finally we have only examined the predictive capacity of this self-report questionnaire with regards to short-term survival in haemodialysis patients and as such, the results should not be extrapolated to other groups of patients with kidney disease, or to the assessment of long-term survival.

## Conclusion

In summary, our self-report comorbidity questionnaire is a simple and reliable tool for obtaining comorbidity data in clinical practice and research studies involving patients with end-stage renal disease on haemodialysis. There is strong agreement between this self-report instrument and data derived from medical records on important comorbid conditions that have an influence on patient outcome. The instrument also provides information on severity of the comorbid diseases. In addition, the comorbidity score generated (CSCS) has comparable predictive power for short-term survival in haemodialysis patients to the CCI. Further work is needed to adapt the questionnaire and to examine its applicability in studies assessing long-term survival and other clinical outcomes in patients with kidney disease.

## Competing interests

The authors declare that they have no competing interests.

## Authors’ contributions

SS: Chief investigator, designed the study and the questionnaire, was involved in patient recruitment, data collection and analysis and wrote the manuscript; JB: involved in patient recruitment and data collection; EV: was involved in designing and supervising the study, reviewing the manuscript and contributing critical intellectual content to the manuscript; JR: was involved in designing and supervising the study, reviewing the manuscript and contributing critical intellectual content to the manuscript; KF: was involved in designing and supervising the study, reviewing the ethics and R&D application, reviewing the manuscript and contributing critical intellectual content to the manuscript. All authors read and approved the final manuscript.

## Pre-publication history

The pre-publication history for this paper can be accessed here:

http://www.biomedcentral.com/1471-2369/15/134/prepub

## Supplementary Material

Additional file 1: Self-report Comorbidity Questionnaire Click here for file
