# Supplementary figures and images for: A self-report comorbidity questionnaire for haemodialysis patients
Source: BMC Nephrol. 2014 Aug 18;15:134. doi: 10.1186/1471-2369-15-134 (PMC4140824; doi:10.1186/1471-2369-15-134)

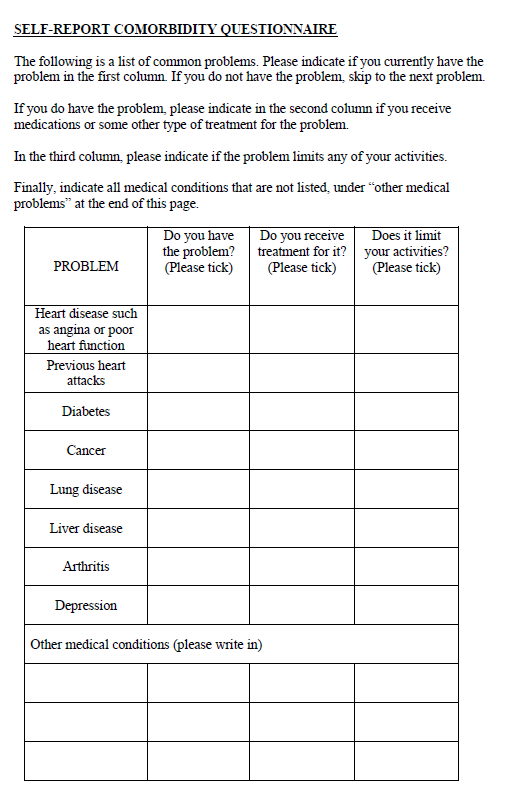

Supplement: Additional file 1: Self-report Comorbidity Questionnaire [file 1471-2369-15-134-S1.png]
